# Connexin 26 in Hearing Health and Disease: StructuralFoundations, Mutation Mechanisms, and Therapeutic Perspectives

**DOI:** 10.3390/ijms27114831

**Published:** 2026-05-27

**Authors:** Weihua Qiu, Kaelah Schneider, Youzhong Guo

**Affiliations:** 1 Department of Medicinal Chemistry, Virginia Commonwealth University, Richmond, VA 23219, USA; 2 Center for Drug Discovery, Virginia Commonwealth University, Richmond, VA 23219, USA; 3 Department of Chemistry, Virginia Commonwealth University, Richmond, VA 23219, USA

**Keywords:** Connexin 26, GJB2, mutations, hearing loss, treatment approaches

## Abstract

Mutations in gap junction protein β-2 (GJB2), encoding Connexin 26 (Cx26), are the most common genetic cause of hearing loss, responsible for up to 50% of inherited non-syndromic cases worldwide. This review covers Cx26 from three perspectives: protein structure, mutant disease mechanisms, and treatment approaches. Structurally, 12 Cx26 subunits assemble into a gap junction channel connecting neighboring cells, enabling exchange of ions and signaling molecules; activity is regulated by calcium, pH, and CO_2_. In the cochlea, Cx26 channels are required for the development of sound-sensing hair cells, maintenance of the electrical gradient needed for hearing, and energy supply during sound processing. GJB2 mutations cause hearing loss through three mechanisms, complete loss of functional protein, failure of channel assembly or membrane delivery, and abnormal channel gating, that damage cochlear cells. Severity ranges from profound congenital deafness to gradual decline, depending on which mutations are inherited. Gene therapy, genome editing, and pharmacological approaches are under investigation; cochlear implantation remains the current standard of care.

## 1. Introduction

Hearing loss is one of the most prevalent chronic sensory deficits worldwide, affecting an estimated 1.57 billion people in 2019—approximately one in five individuals globally—with projections reaching 2.45 billion by 2050 [1,2,3]. Hearing loss is classified etiologically as genetic (at least 60%) or environmental, with genetic forms further divided into syndromic, in which hearing loss accompanies other systemic features, and non-syndromic, which accounts for approximately 70% of genetic hearing loss cases [4,5,6]. Autosomal recessive non-syndromic sensorineural hearing loss (ARNSHL), designated DFNB (Deafness, Familial, Nonsyndromic, autosomal recessive B), represents the most common inheritance pattern (~80% of ARNSHL) and is characterized by severe-to-profound, prelingual hearing loss [7,8,9].

Among the more than 100 genes implicated in hereditary hearing loss, mutations in GJB2 are by far the most prevalent cause of ARNSHL, responsible for up to 50% of cases in many populations worldwide and representing the predominant molecular diagnosis in large, multiethnic comprehensive genetic testing cohorts [6,10,11]. GJB2 encodes Connexin 26 (Cx26), a 26-kDa transmembrane protein that oligomerizes into hexameric hemichannels (connexons), which dock with complementary connexons on adjacent cells to form intercellular gap junction channels [12,13,14,15]. In the cochlea, Cx26 co-assembles with connexin 30 (encoded by GJB6) within two independent gap junction networks—the epithelial cell system and the connective tissue cell system—forming pathways essential for potassium ion (K^+^) recycling, maintenance of the endocochlear potential (+80 to +100 mV), and ATP-mediated intercellular calcium signaling, all of which are indispensable for mechanotransduction [16,17,18,19].

Given the molecular and clinical significance of GJB2-related hearing loss, this review aims to (1) summarize current understanding of Cx26 structure and function; (2) examine the spectrum of GJB2 mutations and their pathogenic mechanisms; and (3) discuss current and emerging therapeutic strategies including cochlear implantation, gene therapy, and pharmacological chaperones. By integrating findings from molecular, cellular, and clinical studies, this review provides a comprehensive and updated overview of Connexin 26-related hearing loss, with particular relevance to the ongoing development of precision therapies.

## 2. The Structure and Function of Connexin 26

Connexin 26 (Cx26), encoded by GJB2, exemplifies the direct relationship between protein architecture and disease pathogenesis. Six subunits assemble into membrane-spanning hemichannels that align between adjacent cells to create complete gap junction channels [20]. These intercellular conduits serve dual critical functions: maintaining cochlear K^+^ homeostasis and metabolic coordination in auditory supporting cells, and synchronizing epidermal differentiation programs [18,21]. With GJB2 variants causing up to 50% of autosomal recessive non-syndromic deafness in certain populations, Cx26 serves as both a therapeutic target and a model for connexinopathies [17,21].

### 2.1. Structure of Cx26

#### 2.1.1. Molecular Architecture of the Connexin 26 Subunit

**Figure 1:**
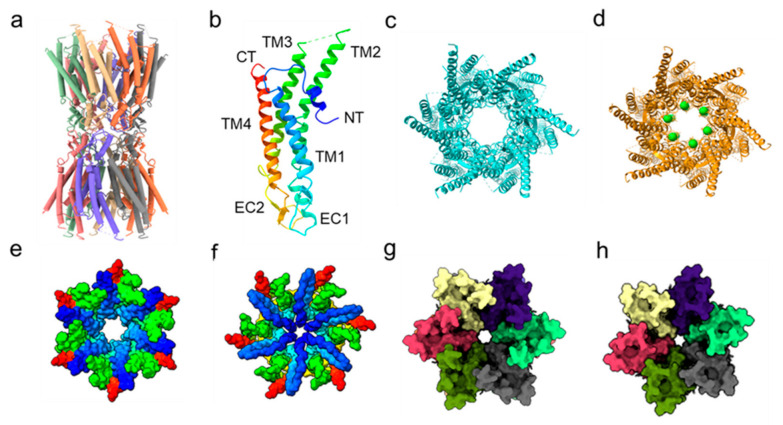
Structures of gap junction channels Cx26 within different environments. (**a**) The architecture of the first high-resolution structure of Cx26 (PDB: 2ZW3), docking two hemichannels. (**b**) One subunit of Cx26 (PDB: 2ZW3) with 4 helixes aligned within the transmembrane regions. EC1 and EC2 are located in the extracellular matrix. The flexible CL loop located between TM2 and TM3 is missing in the structure. The N-terminal region (NT) is inserted into the central pore area. The C-terminal region (CT) is near the extension of TM4. (**c**,**d**) The electrostatic gating mechanism is based on two Cx26 structures with (PDB: 5ER7, colored in brown) or without (PDB: 5ERA, colored in cyan) calcium ions colored in green. (**e**,**f**) The steric “ball-and-chain” mechanism involved in pH regulations during the tissue injury with physiological conditions at pH 7.5 (**e**: PDB: 6UVR, open state) and acidic conditions at pH 6.4 (**f**: PDB: 6UVT, closure state). (**g**,**h**) The conformational changes mechanism involved with CO_2_-induced channel gating at elevated CO_2_ tensions of 55 mmHg CO_2_ (**g**: PDB: 7QER, intermediate closure) and 90 mmHg CO_2_ (**h**: PDB: 7QEQ, substantial constriction). Color coding: panel (**b**), TM1–TM4 in blue, EC1–EC2 in green, NT in red, CT in orange, CL in gray; panels (**c**,**d**), Ca^2+^-bound structure (5ER7) in brown, apo structure (5ERA) in cyan, Ca^2+^ ions in green; Panels (**e**, open state) and (**f**, closed state): color coding by transmembrane helix type, with each helix across all subunits rendered in the same color. Panels (**g**, open state) and (**h**, closed state): subunits are color-coded individually.

Connexin 26 exhibits the canonical connexin topology: four transmembrane helices (TM1–TM4), two extracellular loops (EC1, EC2), and cytoplasmic N-terminal, C-terminal, and loop domains (NT, CT, CL) [12,14,15,22,23]. While membrane and extracellular regions are structurally well-defined through X-ray crystallography and cryo-EM, the dynamic cytoplasmic domains remain incompletely resolved despite their critical roles in channel gating and regulation. The CT sequence, which varies considerably in length and composition across connexin isoforms, drives functional specialization through differential protein-protein interactions and signaling pathway engagement [20]. Six connexin subunits oligomerize into hexameric connexons (hemichannels) within the plasma membrane, creating a central aqueous pore with approximately 1.5 nm diameter [12]. These connexons dock between adjacent cells, forming complete gap junction channels that span the intercellular space (Figure 1a). Connexons may contain identical subunits (homomeric) or mixed isoforms (heteromeric), and docked channels may be homotypic or heterotypic—such as Cx26/Cx30 heteromers in cochlear supporting cells [24] or Cx40/Cx43 heteromers in vascular smooth muscle cells [25]. The compositional variability generates channels with tissue-specific permeability, voltage-gating, and regulatory properties [20], enabling bidirectional exchange of ions, metabolites (≤1.5 kDa), and second messengers.

Individual Cx26 subunits possess a defined molecular architecture essential for functional channel assembly. Four α-helical transmembrane segments (TM1–TM4) arrange counterclockwise when viewed cytoplasmically (Figure 1b), stabilized by intramolecular hydrophobic interactions within subunits and intermolecular contacts between neighboring protomers—both critical for correct folding and hexameric oligomerization [12,14,20,26]. Extracellular loops EC1 and EC2 govern structural stability and hemichannel docking specificity [20,27,28,29,30]. Each extracellular loop (EC1: Cys53, Cys60, Cys65 and EC2: Cys169, Cys174, Cys180) contains three highly conserved cysteine residues that form three interloop intramolecular disulfide bonds—pairing cysteines of EC1 with those of EC2—which together constrain the two loops into a jointly shared antiparallel β-sheet architecture. This cross-loop disulfide scaffold rigidifies the extracellular domain, maintains precise loop topology, and enables accurate hemichannel-to-hemichannel alignment across the ~40 Å intercellular gap during connexon docking [12,20,30,31]. The N-terminal domain adopts an amphipathic helical structure projecting from the cytoplasmic vestibule into the pore lumen, where it executes gating and selectivity functions [12,20]. This conformationally dynamic helix operates as a multimodal sensor, responding to voltage gradients, pH shifts, calcium concentration, CO_2_ levels, and covalent modifications [12,14,15,22,23,32,33]. The cytoplasmic loop (CL, linking TM2–TM3) and C-terminus (CT) exhibit maximal sequence divergence among connexins, determining isoform-specific regulatory profiles [34]. The CL (30–50 residues) mediates pH-dependent gating, scaffolds protein complexes, and modulates channel open probability, though its intrinsic disorder has precluded structural visualization except in recent cryo-EM datasets [12,14,15,20,22,23]. The CT displays extraordinary length variation—from approximately 10 residues in Cx26 to over 150 residues in Cx43 [34]—with Cx26’s minimal CT nonetheless orchestrating regulatory interactions and gating control [12,20]. Importantly, connexin isoforms display selective molecular permeabilities; Cx26 uniquely favors anionic transport, a specialization crucial for cochlear ionic homeostasis [35,36].

#### 2.1.2. Structural Foundations of Regulatory Gating Mechanisms in Cx26

Atomic-resolution structural studies have revolutionized understanding of Cx26 regulatory mechanisms. Since the pioneering 2009 crystallographic analysis, combined X-ray and cryo-EM approaches have resolved Cx26 architecture across multiple functional states, making it the most structurally defined connexin. Table 1 catalogues high-resolution Cx26 structures representing distinct conformational and regulatory configurations [12,14,15,22,23,33].

##### Calcium-Mediated Electrostatic Gating

Maeda et al. [12] obtained the inaugural atomic-resolution structure of human Cx26 at 3.5 Å, fundamentally transforming gap junction structural biology. This breakthrough crystallographic analysis visualized the channel in its open state, revealing molecular-level architecture previously inaccessible through electron microscopy. Critically, the N-terminal helix was observed inserting into the central pore, creating a funnel-shaped constriction that governs pore diameter and molecular selectivity—findings that established the structural framework for all subsequent mechanistic investigations.

Comparative crystallography of Ca^2+^-bound and apo Cx26 (3.29 Å and 3.80 Å, respectively) demonstrated nearly superimposable architectures, excluding substantial conformational closure upon calcium binding (Figure 1c,d) [15]. Instead, Ca^2+^ ions localize at intersubunit interfaces near the extracellular vestibule, where they function electrostatically rather than sterically. This charge-based mechanism—whereby Ca^2+^ coordination establishes a positive electrostatic barrier—selectively impedes cation flux without physical pore occlusion. However, confident assignment of metal ion identity at these resolution limits (3.29–3.80 Å) requires caution: at resolutions above ~2.0 Å, Ca^2+^, Na^+^, and ordered water molecules produce overlapping electron density, and anomalous diffraction experiments or ultra-high-resolution data (ideally ≤1.5 Å) are required for unambiguous coordination geometry assignment. The current Cx26 Ca^2+^ site assignments therefore remain provisional pending validation by higher-resolution or complementary spectroscopic approaches. Additionally, calmodulin may regulate gap junction channels in concert with calcium ions [37,38], though structural characterization of calmodulin-Cx26 complexes is needed to fully define this regulatory axis and its physiological significance in channel gating control.

##### Acidification-Triggered Gating

Direct structural visualization has confirmed the “ball-and-chain” mechanism underlying Cx26 pH sensitivity (Figure 1e,f) [22]. Intracellular acidification drives cytoplasmic domain movements that sterically occlude the channel pore, halting bidirectional exchange of neutral and negatively charged molecules. Cochlear hearing critically depends on precise endolymphatic pH homeostasis (~7.4–7.5), maintained by HCO_3_^−^ secretion via pendrin (SLC26A4) and H^+^ extrusion by the stria vascularis [39,40]. Disruption of this balance—as in Pendred syndrome—causes endolymphatic acidification, collapse of the endocochlear potential, and irreversible hair cell degeneration [19,41]. Notably, cochlear Cx26 channels are themselves pH-gated: endolymphatic acidification can trigger Cx26 closure via the ball-and-chain mechanism, compounding K^+^ recycling failure and cochlear dysfunction [22,42]. Current structural data resolve the occluded state at 7.5 Å [22]; atomic-resolution studies are required to capture the complete conformational rearrangement pathway of the regulatory cytoplasmic domains.

##### CO_2_-Induced Conformational Gating

Ultra-high-resolution cryo-EM analyses (1.9–2.2 Å) across physiologically relevant CO_2_ tensions (PCO_2_ = 20–90 mmHg) revealed that carbon dioxide directly drives N-terminal helix rearrangements that progressively constrict the channel pore (Figure 1g,h) [23]. Mechanistically, CO_2_ may carbamylate Lys125, initiating a cascade of structural changes: cytoplasmic loop reordering, transmembrane domain tilting, and ultimately N-terminal plug insertion that seals the pore [23]. A carbamylation-mimetic K125E substitution produces comparable closure at ~2.4 Å resolution, with cytoplasmic loop stabilization and N-terminal displacement mirroring high-CO_2_ wild-type conformations [14].

In the cochlea, this mechanism carries direct pathophysiological relevance: the Keratitis–Ichthyosis–Deafness (KID) syndrome mutation A88V renders Cx26 hemichannels CO_2_-insensitive and dominantly suppresses wild-type CO_2_ sensitivity, directly linking loss of CO_2_-driven conformational gating to syndromic sensorineural deafness [43]; KID syndrome Cx26 mutations expressed in cochlear organ of Corti supporting cells further produce hyperactive hemichannels with disrupted CO_2_ regulation, confirming physiological CO_2_-mediated gating in the cochlear sensory epithelium [44]. Whether carbamylation-mediated gating operates under normal cochlear homeostasis or exclusively under pathological hypercapnia remains an open question.

##### Future Structural Directions

Despite remarkable progress, current methodologies impose limitations: All published Cx26 structures were determined using detergent-solubilized protein, a necessary compromise that strips the endogenous lipid annulus and may alter the conformational landscape of the NT, CL, and CT domains. Three emerging strategies address this limitation. Styrene-maleic acid (SMA) copolymers extract membrane proteins directly from native membranes, encapsulating them together with boundary lipids in polymer-stabilized nanodiscs (SMALPs) without detergent exposure [45,46]; however, SMA sensitivity to divalent cations constrains its compatibility with Ca^2+^-dependent gating studies. MSP-based reconstituted nanodiscs permit precise lipid composition control and have already enabled 1.9 Å resolution structures of the closely related Cx46/50 channel [47], establishing proof of principle for Cx26. Nascent methods like native cell membrane nanoparticles show potential for preserving endogenous lipid compositions but require systematic validation [48,49]. Achieving truly native-like structures through detergent-free isolation, time-resolved cryo-EM capturing gating intermediates, and integrative biophysical validation will be crucial to delineate lipid-dependent conformational landscapes and establish definitive physiological gating mechanisms and connexinopathies.

The unresolved NT, CL, and CT domains represent compelling targets for AI-assisted integrative modeling. AlphaFold (AF)3’s diffusion-based architecture reduces hallucinations in disordered regions compared to AF2 [50,51], enabling ensemble models of Cx26 cytoplasmic domains that generate testable conformational hypotheses. RoseTTAFold All-Atom (RFAA) further enables joint modeling of proteins with metals, covalent modifications, and small molecules [52]—predicting Ca^2+^ coordination, CO_2_ carbamylation of Lys125, and calmodulin interfaces in a single framework. Critically, both tools may model the structural consequences of GJB2 pathogenic mutations, providing atomic-level insight into how specific alleles disrupt gating. Experimental validation through HDX-MS, crosslinking mass spectrometry, and SAXS integrated within IMP [53] would yield the first complete structural model of the Cx26 connexon—a foundation for rational discovery of small-molecule modulators targeting GJB2-related hearing loss.

### 2.2. Cellular Distribution and Function of Connexin 26 in the Cochlea

#### 2.2.1. Cellular Distribution of Connexin 26

**Figure 2:**
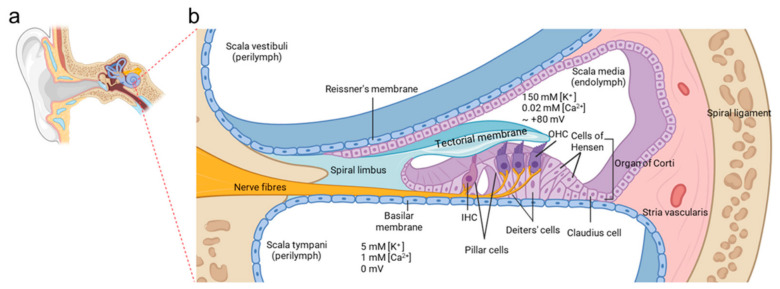
Schematic diagrams of cochlear anatomy with ions circulations. (**a**) The ear anatomy of human body. (**b**) The anatomy of cochlear association with potassium recycling and Connexin 26 expression in different cells. IHC: Inner hair cells; OHC: Outer hair cells. Created in Biorender. Qiu W, 2026 https://app.biorender.com/citation/6949a45e0f17651f9c095487, licensed under the Creative Commons Attribution 4.0 International License (CC BY 4.0). Adapted from references [17,21].

The cochlea transduces mechanical vibrations into electrochemical signals through specialized cellular architecture (Figure 2) [54]. Cx26 displays spatially segregated expression across two distinct yet interconnected gap junction networks critical for auditory processing [55,56,57]. The epithelial network spans organ of Corti supporting cells—including inner/outer sulcus, border, phalangeal, pillar, Deiters’, Hensen’s, and Claudius’ cells—with differential connexin distribution: Cx30 dominates in Deiters’ cells while Cx26 predominates peripherally in Hensen’s and Claudius’ cells [58,59,60]. The connective tissue network comprises stria vascularis intermediate and basal cells, spiral ligament fibrocytes (types I, II, V), and spiral limbus interdental cells (Figure 2b) [60,61,62]. Notably, sensory hair cells completely exclude connexin expression, functioning independently of gap junctional coupling [62,63]. This compartmentalized distribution underlies Cx26’s stage-specific roles: developmental ATP-Ca^2+^ signaling cascades, mature cochlear K^+^ homeostasis and metabolic coordination, and protection against age-related auditory decline.

#### 2.2.2. Developmental Roles: ATP-Calcium Signaling and Cochlear Morphogenesis

Before hearing onset, Cx26 orchestrates ATP-Ca^2+^ signaling cascades essential for sensory epithelium maturation and structural development [16,64]. Cx26 ablation disrupts intercellular Ca^2+^ wave transmission, producing abnormal apoptosis, ATP depletion, and failure of inner hair cell functional maturation—a non-cell-autonomous mechanism whereby supporting cell connexin deficiency impairs ribbon synapse formation and ion channel development in hair cells that themselves lack connexin expression [16,17,64]. The defining pathological hallmark involves failure of the tunnel of Corti to open around postnatal day 6 (P6), blocking perilymph access and disrupting ionic homeostasis, followed by progressive tissue degeneration initiated in Claudius cells around P8 and spreading to outer hair cells by P13 [18,65,66]. Temporal deletion experiments establish a critical developmental window before postnatal day 10: early ablation (P0–P4) produces severe congenital deficits, while delayed deletion (P8–P10) yields significantly attenuated phenotypes [18,67]. These coordinated developmental programs establish the architectural and physiological foundation required for mature cochlear function and hearing acquisition.

#### 2.2.3. Mature Cochlear Function: Ionic Homeostasis, Metabolic Coupling, and Active Amplification

In the mature cochlea, Cx26-containing gap junction networks maintain ionic balance, coordinate metabolic processes, and support active sound amplification [18,35,59,68]. The established K^+^ recycling model proposes that gap junctions mediate spatial buffering, transporting K^+^ from hair cell basolateral surfaces through supporting cells and fibrocytes to the stria vascularis for endolymphatic re-secretion via voltage-gated potassium channel (KCNQ1/KCNE1), sustaining the endolymphatic K^+^ concentrations (150 mM) and endocochlear potential (+80 mV) required for mechanotransduction [69,70,71,72]. Concurrently, the avascular sensory epithelium relies on gap junction-mediated metabolic networks for distributing glucose and ATP, with connexin ablation producing mitochondrial impairment, energy depletion, and oxidative damage [18,35,73]. Adult-onset connexin deficiency reduces distortion product otoacoustic emissions—markers of active cochlear amplification—despite hair cells lacking connexin expression, demonstrating that gap junctions indirectly modulate outer hair cell electromotility through ionic buffering, metabolic provision, or paracrine signaling mechanisms [68,74].

Taken together with the developmental evidence in Section 2.2.2, these findings support an integrated, temporally staged model of Cx26-dependent cochlear pathogenesis (Figure 2). Prior to hearing onset, ATP–Ca^2+^ signaling is the dominant requirement, accounting for the profound congenital deafness of null GJB2 alleles through mechanisms independent of endocochlear potential [16,17,18,64,65,66,67,75]. After hearing onset, K^+^ spatial buffering and endolymphatic homeostasis become principal, underpinning progressive and adult-onset phenotypes [68,69,70,71,72,76]. Metabolic coupling operates across both windows, amplifying dysfunction through energy depletion and oxidative stress [18,35,73]. This framework directly informs therapeutic timing: Cx26 restoration must occur within the critical developmental window to prevent irreversible morphogenetic failure [77,78,79,80].

#### 2.2.4. Age-Related Functions: Cx26 Degradation and Presbycusis

Progressive Cx26 decline represents an emerging mechanism underlying age-related hearing loss (ARHL) [73,74,81,82]. Aged cochleae show reduced Cx26 expression and fragmented gap junction plaques, degraded through dynamin-2-dependent endocytosis and lysosomal pathways [73,82,83]. Adult-onset conditional knockout models confirm causality directly: they recapitulate progressive high-frequency hearing loss, outer hair cell degeneration, and spiral ganglion neuron demyelination—the hallmark features of presbycusis [73]. The underlying mechanism centers on a convergent pathway of oxidative stress, reduced glutathione release, and dysregulated Nrf2 antioxidant signaling [18,74]. Independently, age-related GJB2 promoter hypermethylation silences Cx26 expression at the epigenetic level, adding a second layer of age-dependent decline [81]. The clinical impact extends beyond presbycusis. Among patients carrying GJB2 mutations, 19–28% exhibit late-onset progressive hearing loss rather than purely congenital phenotypes, confirming that Cx26 insufficiency drives audiological deterioration across the lifespan [77,84]. This positions early Cx26 degradation as both a mechanistic driver and a candidate biomarker for ARHL onset and progression [73,82]. Collectively, integrating developmental, physiological, and aging functions of Cx26 is essential for designing stage-specific therapeutic strategies: early gene therapy combined with anti-inflammatory agents for congenital cases, pharmacological channel modulators or antioxidants for progressive loss, and interventions targeting age-related protein degradation for presbycusis prevention [11,73,78,79,81,82,85,86,87,88,89].

## 3. Pathogenic Mechanisms of Cx26 Mutations

### 3.1. Types of Mutations and Major Mutants

Mutations in GJB2, the gene encoding Connexin 26 (Cx26), constitute the predominant genetic etiology of DFNB1, responsible for approximately 50% of hereditary deafness cases globally [11]. More than 400 distinct pathogenic variants have been documented, encompassing missense, nonsense, frameshift, and splice-site alterations that produce a broad audiological spectrum ranging from mild impairment (26–40 dB hearing level) to profound deafness (>90 dB HL) [7,11,78]. Mutation spectra vary substantially across populations due to founder effects and population-specific evolutionary dynamics influencing variant distribution [90,91,92,93,94,95]. Variant classification follows the ACMG/AMP framework [96]; GJB2-specific refinements were established by the ClinGen Hearing Loss Variant Curation Expert Panel (HL-VCEP), whose Version 2 specifications (2022) introduced gene-specific adjustments to allele-frequency thresholds, functional evidence weighting, and de novo criteria, substantially reducing GJB2 variants of uncertain significance in ClinVar and providing the authoritative reclassification of the contested p.Met34Thr and p.Val37Ile variants as pathogenic with incomplete penetrance [97].

#### 3.1.1. Missense Mutations

Missense variants constitute approximately 60% of pathogenic GJB2 alterations, introducing amino acid substitutions that compromise protein folding, gap junction oligomerization, membrane trafficking, or channel conductance [13,98]. The V37I (c.109G > A) substitution in transmembrane domain 1 predominates in East Asian populations (allele frequencies reaching 7.7% in Taiwan), producing mild-to-moderate, late-onset progressive auditory decline characterized by incomplete penetrance, disrupted gap junction plaque morphology, and threshold deterioration averaging ~1 dB annually [99,100,101]. In contrast, the R75W (c.223C > T) variant in extracellular loop 1 abolishes pore conductance, causing autosomal dominant hearing loss (DFNA3) via dominant-negative interference with wild-type channels [102,103,104]. Contemporary genomic surveys have identified additional GJB2 variants with population-specific distributions and variable phenotypic impact, including p.I203T—predominantly found in East Asian populations and recently associated with more severe hearing loss in compound carriers—alongside p.M34T, prevalent in European populations and typically linked to mild-to-moderate hearing loss, and p.W44C, a rare dominant variant restricted to Northern European-descended families, causing progressive hearing loss [11,78,91,99,105,106,107]. Notably, machine learning algorithms developed in 2024 now enable longitudinal progression forecasting for GJB2-related hearing loss, with particular accuracy for V37I carriers [108].

#### 3.1.2. Nonsense Mutations

Nonsense variants introduce premature termination codons that generate truncated polypeptides rapidly eliminated through nonsense-mediated mRNA decay surveillance pathways, invariably producing severe-to-profound congenital deafness due to complete loss of functional protein [96]. The E47X (c.139G > T) variant exemplifies this class, frequently occurring in compound heterozygous configurations alongside frameshift alleles, resulting in profound bilateral congenital auditory loss with absent auditory brainstem responses [109,110]. Additional nonsense alleles documented across diverse populations include W77X (c.231G > A), Q7X (c.19C > T), and Q57X (c.169C > T), each ablating essential structural domains—transmembrane segments, extracellular loops, or cytoplasmic regions—critical for connexon oligomerization, hemichannel trafficking, and functional gap junction channel assembly [109,110,111,112,113].

#### 3.1.3. Frameshift Mutations

Insertions or deletions lacking triplet divisibility generate frameshift variants that alter downstream codon sequences, introducing premature stop signals and yielding truncated nonfunctional proteins that manifest as severe-to-profound prelingual deafness [78]. Among these, c.35delG (p.Gly12ValfsTer2) represents the most frequently encountered variant worldwide, constituting 50–80% of pathogenic GJB2 alleles in populations of European ancestry where carrier rates reach 0.8–0.9%; nonetheless, emerging clinical data reveal rare instances of atypical presentations featuring asymmetric hearing profiles or delayed progressive onset [92,114,115,116]. By contrast, East Asian populations exhibit c.235delC as the predominant pathogenic variant, accounting for approximately 57% of disease-causing GJB2 alleles with carrier prevalence near 1.89%, patterns consistent with strong founder effects [99,117,118,119,120,121]. Ethnically enriched frameshift variants include c.167delT in Ashkenazi Jewish lineages, c.299_300delAT across multiple Asian populations, and the splice-junction variant IVS1 + 1G > A (c.-23 + 1G > A) prevalent throughout Russian and Siberian regions, reflecting localized founder phenomena [101,122,123,124,125,126,127]. Ongoing genetic surveillance continuously uncovers additional rare variants, including c.188delT, identified within Chinese kindreds [128].

#### 3.1.4. Genotype-Phenotype Correlations and Population Variation

Null alleles typically manifest as severe auditory phenotypes, whereas compound heterozygous genotypes pairing one truncating allele with one hypomorphic (partial-function) variant often yield milder, progressive hearing decline [11,78,129]. Groundbreaking 2023 research has fundamentally revised understanding of heterozygote carriers: Cx26^+^/^−^ individuals—representing approximately 10–20% of the global population—exhibit paradoxical auditory hypersensitivity resembling hyperacusis, enhanced cochlear amplification mechanisms, and substantially elevated susceptibility to noise-induced damage [57,130]. Phenotypic expression variability in GJB2-associated hearing loss reflects complex interactions between genetic modifiers and environmental exposures [131]. Geographic mutation profiles demonstrate marked heterogeneity: c.35delG accounts for 50–80% of Caucasian pathogenic alleles [116,132]; c.235delC predominates in East Asian populations (57% in Taiwanese cohorts) [100,101,120]; V37I reaches the highest frequencies across East Asian populations, correlating with milder audiological outcomes; African-ancestry populations display proportionally lower GJB2 contributions (~10% versus 30–50% in non-African populations) [133,134]. Large-scale neonatal genetic screening initiatives validating these ethnic-specific distributions underscore the necessity for ancestry-informed diagnostic protocols optimizing detection sensitivity [135,136,137,138]. From a clinical management perspective, heterozygous Cx26^+^/^−^ carriers warrant audio-logical surveillance: in addition to recurrence-risk counseling, the recently demonstrated noise-vulnerability in Cx26^+^/^−^ individuals [57,130], suggests that occupational and recreational noise exposure limits should be discussed during carrier consultations arising from family or newborn screening cascade testing [135,136,137,138].

**Figure 3:**
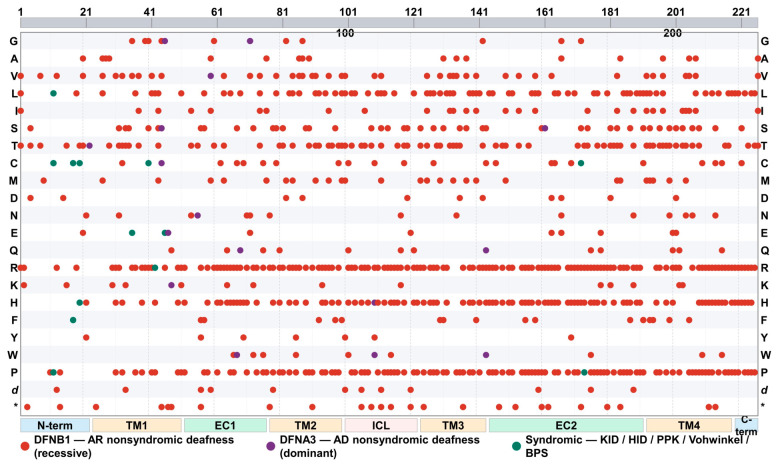
Deafness-associated mutation sites in the GJB2 gene product Connexin-26 (UniProt: P29033). The 226-amino acid sequence of Connexin-26 (Cx26) is displayed along the *x*-axis, with each row on the *y*-axis representing a possible amino acid substitution (single-letter code). Each dot indicates a confirmed pathogenic mutation at that position, color-coded by inheritance pattern and clinical phenotype: red dots, DFNB1; purple dots, DFNA3; green dots, syndromic hearing loss associated with skin disorders (keratitis–ichthyosis–deafness [KID] syndrome, hystrix-like ichthyosis with deafness [HID], palmoplantar keratoderma [PPK], Vohwinkel syndrome, or Bart-Pumphrey syndrome [BPS]). The bottom row labeled “*” represents nonsense (stop codon) mutations; the row labeled “d” represents frameshift mutations caused by small insertions or deletions. The colored bar beneath the plot indicates the domain architecture of Cx26: N-terminal domain (N-term), four transmembrane domains (TM1–TM4), two extracellular loops (EC1 and EC2), the intracellular cytoplasmic loop (ICL), and the C-terminal domain (C-term). Mutation data were compiled from UniProt P29033 (https://www.uniprot.org/uniprotkb/P29033, accessed on 19 April 2026), references from [85,91,103,109,126,139,140,141,142,143,144,145], and publicly available variant databases including ClinVar and the Human Gene Mutation Database (HGMD).

The mutational landscape of GJB2 is remarkably broad, with over 400 confirmed pathogenic variants spanning the entire 226-amino acid Cx26 sequence (Figure 3). Recessive DFNB1 variants distribute throughout all structural domains, while dominant and syndromic mutations cluster preferentially in the N-terminal domain and EC1, foreshadowing their distinct molecular mechanisms discussed in Section 3.2. Despite this extensive catalog, the current map remains open, as most genomic studies have focused on European and East Asian populations. Ongoing large-scale whole-genome sequencing across globally diverse cohorts is expected to substantially expand the known spectrum of pathogenic GJB2 variants in the coming years.

### 3.2. Effects on Protein Function

Pathogenic Cx26 mutations exert their deleterious effects through multiple distinct molecular mechanisms: disrupted intracellular trafficking and plasma membrane insertion, impaired oligomerization into hexameric connexon structures, aberrant channel conductance properties and regulatory gating behavior, and toxic gain-of-function activity [13,55,103]. Understanding the specific mechanism underlying each mutation enables mutation-tailored therapies and accurate genotype-based prognosis [11].

#### 3.2.1. Trafficking, Oligomerization, and Functional Grouping

Systematic functional analyses reveal that mutations in different protein domains cause defects at specific stages of channel formation [13]. This creates two distinct failure patterns: mutations in transmembrane domains (TM1/TM2) typically prevent the protein from reaching the cell membrane or assembling properly (Group 3 “trafficking-deficient”), while mutations in extracellular loop 1 (EC1) allow normal trafficking and assembly but produce channels that don’t work correctly (Group 2 “assembled but nonfunctional”) [55,103,146]. Classic examples illustrate this pattern: M34T reaches the membrane but cannot form hexamers efficiently, W77R gets trapped inside the cell and cannot assemble at all, while W44C successfully traffics and assembles but fails to transfer molecules between cells [146]. Studies in cochlear cells confirmed these two distinct failure modes [55]. Molecular simulations explain why transmembrane mutations cause these trafficking problems—they destabilize the protein structure and create barriers that block normal channel function [147]. In contrast, EC1 mutations work through a different mechanism: they alter a critical pore region (position 49) that controls which molecules can pass through the channel, which explains why Cx26 and Cx30 have different permeability despite similar structures [24,148]. Understanding whether a mutation causes trafficking failure versus functional failure is critical for designing appropriate treatments—trafficking defects may require cellular correction strategies, while functional defects may need approaches to restore channel activity [55,103,146].

#### 3.2.2. Altered Channel Permeability and Gating

GJB2 missense mutations disrupt channel function by altering pore structure, electrical gating, and ion selectivity [24,33,148,149]. Mutations affect channels differently depending on their location. Extracellular loop mutations illustrate two distinct mechanisms: D50N weakens calcium-dependent closure, shifts voltage gating, and reduces conductance by disrupting stabilizing interactions at the pore entrance [148,149], while G45E introduces negative charge that hyperactivates channels, increases calcium permeability, and causes cell death [150,151]. A single amino acid change at pore position 49 can completely reverse whether the channel prefers positive or negative ions, explaining functional differences between Cx26 and Cx30 [24,148]. Transmembrane domain mutations work differently—they destabilize the pore structure itself, forcing channels into low-conductance states with abnormal voltage responses [33]. Critically, Cx26 gap junctions are much more permeable to ATP than either Cx26 hemichannels alone or Cx30 channels [73,151]. This means mutations may appear benign in hemichannel assays, but severely impair energy molecule transfer in actual cochlear tissue where channels connect cells—directly explaining how permeability defects cause hearing loss.

#### 3.2.3. Gain-of-Function and Hemichannel Dysfunction

Multiple GJB2 variants cause disease by creating hyperactive hemichannels that remain abnormally open, leading to excessive calcium influx, ATP leakage, and cell death in cochlear and skin cells. These mutations disrupt different regulatory mechanisms depending on their location. The D50N/Y mutation in extracellular loop 1 weakens calcium-dependent closure, producing constitutively open channels [152,153]. The N-terminal mutations I30N and S17F fail to form normal gap junctions but generate toxic hemichannels: I30N causes Golgi retention and aberrant hemichannel activity, elevating intracellular calcium in a manner reversible by the hemichannel blocker carbenoxolone [153], while S17F creates hyperactive hemichannels in cochlear organ of Corti supporting cells that produce hair cell stereocilia loss, with hyperactivity dependent on co-expression with wild-type connexins including Cx30 [44]. The A40V mutation disrupts pH and zinc regulation, preventing normal channel closure [154], whereas G12R produces excessive activity and cell death that can be partially reduced by raising extracellular calcium [155]. Collectively, mutations across the N-terminus (S17F, I30N), transmembrane/loop interface (A40V), and extracellular loop (D50Y, G12R) all produce gain-of-function toxicity by impairing the normal regulatory mechanisms—calcium, pH, zinc, or voltage gating—that keep hemichannels closed, directly linking aberrant channel activity to hearing loss and skin disorders.

#### 3.2.4. Impact on Cochlear Ion Homeostasis and K^+^ Recycling

Gap junction networks containing Cx26 form continuous connections between cochlear support cells that recycle potassium ions from sensory hair cells back to the endolymph, maintaining the electrical potential (~+80 mV) necessary for hearing; loss of Cx26 disrupts this potassium recycling, reducing the electrical gradient and impairing auditory function [18,81,156]. Heteromeric Cx26/Cx30 gap junctions accelerate intercellular Ca^2+^ wave propagation and metabolic coordination across the organ of Corti, reinforcing ionic homeostasis [157]. Importantly, Cx26 channels are much more permeable to ATP and negatively charged molecules than either Cx26 hemichannels alone or Cx30 channels, making Cx26 uniquely critical for maintaining the ionic and energy balance required for hair cell survival [73]. Studies in animal models demonstrate that reduced or degraded Cx26 increases vulnerability to noise damage, persistently lowers the endocochlear potential, and accelerates degeneration of both hair cells and auditory neurons—directly linking Cx26 dysfunction to failed potassium recycling and long-term hearing loss [81,158].

As elaborated in the integrated model proposed in Section 2.2.3, K^+^ recycling disruption represents one component of a temporally staged pathogenic cascade; its relative contribution to hearing loss severity is greatest in mature-onset cases, whereas developmental ATP–Ca^2+^ signaling failure is the primary determinant of profound congenital phenotypes [18,65,75,76].

## 4. Current and Emerging Therapeutic Strategies

### 4.1. Cochlear Implantation

GJB2-related hearing loss currently lacks a curative intervention, with cochlear implantation remaining the only established clinical management option—one that achieves excellent speech and language outcomes, particularly with early implantation before age 3.5 years, owing to the preserved spiral ganglion architecture characteristic of DFNB1, even still with some challenges [6,11,159,160,161]. Nevertheless, known limitations include substantially impaired music perception due to the coarse spectral resolution of electrode arrays (12–22 channels), poor bilateral synchronization limiting spatial hearing and sound localization in noise, and long-term dependence on external hardware with inequitable access across healthcare systems [6,11,159,160,161].

### 4.2. Emerging Therapeutic Strategies

#### 4.2.1. AAV-Based Gene Therapy

Gene therapeutic strategies are currently under preclinical investigation for GJB2-related hearing loss. AAV-mediated GJB2 gene replacement has advanced through successive preclinical studies. The first demonstration came from Yu and colleagues, who showed that viral Cx26 delivery into conditional Gjb2 knockout mice restored gap junction network integrity and reduced cochlear cell death, though hearing thresholds were not significantly improved [162]. This foundation was built upon by Sun and coworkers, whose co-injection of AAV1 and the inner-ear-tropic AAV-ie serotype—both carrying Gjb2 under the supporting-cell-specific SCpro promoter—restored hearing in two Gjb2-deficient mouse models with no toxicity in cynomolgus monkey cochleae [163]. A subsequent study by Wang and colleagues further refined this approach by combining AAV-mediated Cx26 delivery with dexamethasone, reducing inner ear inflammation and outer hair cell loss [80]. Working in parallel, Ivanchenko and coworkers applied ATAC-seq chromatin profiling to identify gene regulatory elements that restrict GJB2 expression to the correct cochlear cell types; AAV vectors carrying these elements prevented cochlear degeneration and rescued hearing in conditional Gjb2 knockout mice, with no off-target expression detected in cynomolgus monkey cochleae [86].

The preclinical progression of GJB2 gene therapy has relied on a hierarchy of animal models, each contributing distinct and complementary evidence (Table 2). Mouse models have provided foundational proof of concept for AAV-mediated hearing rescue but are limited by rapid cochlear degeneration and anatomy that diverges substantially from humans. Porcine and non-human primate models address translational gaps in cochlear size, cell-type composition, and surgical accessibility, and are increasingly required by regulatory agencies prior to clinical trials [80,85,86,162,163,164,165].

#### 4.2.2. CRISPR-Based Gene Editing

CRISPR-based gene editing represents a promising approach to correct dominant-negative GJB2 mutations, which cannot be treated by gene replacement alone. The R75W mutation—a single DNA letter change (C-to-T) that causes both hearing loss and palmoplantar keratoderma—fragments gap junction plaques in cochlear supporting cells. Ukaji and colleagues engineered a miniaturized adenine base editor (SaCas9-NNG-ABE8e) small enough to fit within a single AAV vector, which was delivered to inner ear cells using the AAV-Sia6e serotype. This all-in-one vector precisely corrected the mutation (converting T back to C) in human cells and, in a transgenic mouse model carrying the R75W mutation, restored fragmented gap junction plaques to normal, orderly structures in cochlear supporting cells [164]. Because it corrects a single DNA letter rather than replacing the entire gene, this strategy is applicable to other dominant GJB2 mutations and related connexin disorders such as keratitis–ichthyosis–deafness syndrome.

Notwithstanding this therapeutic promise, rigorous safety evaluation remains a prerequisite for clinical translation of CRISPR-based inner ear therapies. Off-target editing—wherein the Cas9–guide RNA complex modifies unintended genomic loci—represents the foremost genotoxicity concern. A tiered orthogonal assessment strategy is now standard practice in the field: computational prediction tools such as CasOFFinder first enumerate candidate off-target sites genome-wide based on sequence similarity and mismatch tolerance [166]; these predictions are subsequently validated by unbiased empirical assays including GUIDE-seq, which captures double-strand break sites in living cells via incorporation of a short donor oligonucleotide, and CIRCLE-seq, a highly sensitive in vitro method that identifies cleavage events across the entire genome without prior site nomination [167,168]. For the SaCas9-NNG-ABE8e construct employed in the GJB2 R75W correction study [164], application of this workflow is particularly warranted: ABE8e is a highly efficient but comparatively promiscuous deaminase variant, and comprehensive off-target profiling using CHANGE-seq-BE or analogous base-editor-specific assays has revealed that ABE8e off-target sites substantially exceed those of the parent Cas9 nuclease, underscoring the need for tool-specific rather than nuclease-centric safety assessment [169]. A related and clinically consequential concern is bystander editing, in which adenines within the ABE editing window adjacent to the target base undergo unintended A-to-G conversion. In the cochlear context, bystander edits at conserved residues flanking the R75W locus could introduce secondary amino acid substitutions in Cx26, potentially disrupting channel function or oligomerization; ABE9 and other next-generation narrow window variants have been developed specifically to minimize this risk and should be evaluated as alternatives in future iterations [170]. Finally, long-term in vivo follow-up remains critically undercharacterized for inner ear gene editing. The post-mitotic nature of cochlear supporting cells reduces concerns of clonal expansion from oncogenic off-target hits, yet sustained expression of AAV-delivered base editors over months to years raises the possibility of cumulative off-target accumulation. Future studies should incorporate whole-genome sequencing of cochlear tissue at extended timepoints, auditory brainstem response monitoring beyond the currently reported short-term windows, and assessment of immune responses to bacterial-derived Cas9 orthologs, to establish the comprehensive safety profile required for regulatory approval of CRISPR-based hearing loss therapies.

#### 4.2.3. Antisense Oligonucleotides

Antisense oligonucleotides (ASOs)—chemically modified single-stranded nucleic acids that modulate gene expression through RNase H-mediated degradation, splice-switching, or translational blockade—represent a complementary platform to AAV-based strategies. For dominant-negative GJB2 mutations (R75W, W44C), allele-specific ASOs selectively suppress the mutant transcript while preserving the wild-type allele, as demonstrated by >70% selective knockdown of GJB2 R75W in a mouse model without affecting endogenous Gjb2 expression [171]. For splice-site variants such as IVS1 + 1G > A, splice-switching ASOs redirect aberrant pre-mRNA processing toward productive exon inclusion, a mechanism with strong cochlear validation in Usher syndrome models via round window membrane delivery [172,173]. The most recent demonstration of allele-preferential ASOs targeting dominant-negative KCNQ4 p.W276S via round window injection further confirms the feasibility of this delivery route for cochlear supporting cells, hair cells, and spiral ganglion neurons [174]. Phosphorothioate-backbone ASOs with 2′-O-methoxyethyl or LNA modifications provide the chemical stability required for sustained activity in the post-mitotic cochlear epithelium [175]. Key challenges—delivery timing, cell-type-specific dosing, and duration of effect—remain to be defined in GJB2 preclinical models.

### 4.3. Pharmacological Chaperones

Pharmacological strategies targeting defective Cx26 membrane trafficking constitute a non-surgical approach, particularly for GJB2 missense variants encoding structurally intact but mistrafficked protein: chemical chaperones, including VRT-534, restore mutant Cx26 channel function in cochlear-relevant cell models [88]; and small molecules promoting TM4-domain mutant membrane localization have achieved hearing recovery and reduced hair cell degeneration in vivo, validating membrane-trafficking rescue as a tractable therapeutic axis [87].

## 5. Summary

This review has examined Connexin 26 (Cx26), encoded by GJB2, as the central molecular determinant of the most common form of hereditary hearing loss. High-resolution crystallography and cryo-EM have defined how four transmembrane helices per subunit scaffold hexameric connexon assembly and intercellular docking, with channel gating regulated by Ca^2+^, intracellular pH, and CO_2_ through mechanistically coupled conformational transitions. Resolving the calmodulin–Cx26 complex structure remains a critical unmet need, as it would define the Ca^2+^-sensing binding interface and clarify how calmodulin modulates NT and CL domain positioning during gating. Within the cochlea, Cx26 fulfills indispensable stage-specific roles: during development, it orchestrates ATP–Ca^2+^ signaling essential for inner hair cell maturation and organ of Corti morphogenesis; in the mature organ, it sustains K^+^ recycling, the endocochlear potential, and gap junction-coupled metabolic networks supporting active amplification; and during aging, progressive Cx26 degradation extends connexin pathophysiology to late-onset progressive decline. GJB2 variants act through three mechanistic routes—loss-of-function via nonsense-mediated decay, dominant-negative impairment of oligomerization or trafficking, and toxic gain-of-function through hyperactive hemichannels—producing an audiological spectrum shaped by allele combination, founder effects, and dosage-sensitive vulnerability in heterozygous carriers. Translating this mechanistic understanding into developmentally timed gene replacement, base editing, and ASO strategies represents the central therapeutic challenge of the next decade.

## Figures and Tables

**Table 1:** High-resolution structures of Connexin 26 deposited in the Protein Data Bank.

PDB/ EMDB	Method	Resolution (Å)	State/Condition	Key Features	Reference
2ZW3	X-ray	3.50	Open, physiological pH	First atomic structure; dodecameric architecture	[12]
5ERA	X-ray	3.80	Apo (no Ca^2+^)	Comparison structure for calcium gating	[15]
5ER7	X-ray	3.29	Ca^2+^-bound (closed)	Electrostatic gating mechanism; 12 Ca^2+^ ions	[15]
6UVR	Cryo-EM	4.00	Physiological pH (7.5)	Open state; NT ball-and-chain gating	[22]
6UVS	Cryo-EM	4.20	Low pH (6.4)	Open state; NT ball-and-chain gating	[22]
6UVT	Cryo-EM	7.50	Low pH (6.4)	Closed state; NT ball-and-chain gating	[22]
EMD-22789	Cryo-EM	4.20	N176Y mutant	Open state; Hemichannel	[33]
7QET	Cryo-EM	2.10	20 mmHg PCO_2_	Physiological CO_2_; baseline open conformation	[23]
7QER	Cryo-EM	2.20	55 mmHg PCO_2_	Intermediate closure; progressive pore narrowing	[23]
7QEQ	Cryo-EM	1.90	90 mmHg PCO_2_	Substantial constriction; coupled conformational changes	[23]
8Q9Z	Cryo-EM	2.40	K125E mutant	Mimics carbamylation; constitutively closed; CL resolved	[14]

**Table 2:** Comparative summary of animal models used in Cx26/GJB2 gene therapy research. HC = hair cell; GJ = gap junction; KO = knockout; ABR = auditory brainstem response.

Species	Model/Mutation	AAV Serotype(s)	Key Outcomes & Hearing Rescue	Translational Role & Cochlear Anatomy	Key Ref.
Mouse (*Mus musculus*)	Conditional Gjb2 KO (Sox10-Cre; Sox10iCreERT2)	AAV1, AAV-ie; AAV2.7m8	GJ network rescue; HC preservation & ABR improvement; note: restored GJ coupling only, no ABR rescue	Primary efficacy model; narrow therapeutic window; small cochlea & rapid HC loss limit translational relevance	[77,148,149]
Mouse (*Mus musculus*)	R75W dominant-negative transgenic (Syndromic KID/PPK)	AAV-Sia6e (SaCas9-NNG-ABE8e)	GJ plaque structural restoration in cochlear supporting cells; ABR not reported	Dominant/syndromic mutation correction model; base-editing proof of concept; poor anatomical match	[78]
Pig (*Sus scrofa*; Bama miniature pig)	Unconditional GJB2 KO (CRISPR/Cas9); AAV cochlear transduction	Combined AAV (AAV1 + AAV-ie)	Profound congenital deafness; HC depletion; AAV transduction confirmed; efficacy pending	Emerging translational platform; large cochlea; congenital phenotype replicates human DFNB1; closest anatomical model to human	[149,150]
Cynomolgus monkey (*Macaca fascicularis*)	Wild-type cochlea — safety/tropism assessment only (no GJB2 KO model)	AAV-ie; AAV-GRE	No hearing impairment; negligible systemic toxicity; cell-specific GJB2 expression confirmed	Regulatory safety step before clinical translation; excellent human-like cochlear anatomy & cell types	[79,149]

## Data Availability

No new data were created or analyzed in this study.
